# Spontaneous bilateral hemothorax in neurofibromatosis type 1 due to internal thoracic artery aneurysm: Case report

**DOI:** 10.1016/j.ijscr.2020.02.026

**Published:** 2020-02-19

**Authors:** Antonio Felipe Neto, Edson Gonçalves Ferreira, Larissa de Melo Freire Golveia Silveira, Filipe Gusmão, Karen Ruggeri Saad, Paulo Fernandes Saad

**Affiliations:** a Escola Superior de Ciências da Saúde, Edifício Fepecs - SMHN Quadra 03, Conjunto A, Bloco 1 - Asa Norte. Brasília, DF, 70710907, Brazil; b Universidade Federal do Vale do São Francisco, Av. Jose de Sá Maniçoba, s/n Centro. Petrolina, PE, 56304917, Brazil

**Keywords:** Hemothorax, Neurofibromatosis 1, Aneurysm, Diagnosis, Differential

## Abstract

**Introduction:**

Vascular lesions are associated with neurofibromatosis 1, including stenosis and aneurysms.

**Presentation of a case:**

A 43-year-old man presented with sudden respiratory failure in our emergency medical service. Physical examination suggested diagnosis of neurofibromatosis. Chest computed tomography revealed a sizeable bilateral hemothorax. He was then submitted to arteriography, which evidenced the right internal thoracic artery aneurysm. The aneurysm was selectively catheterized and embolized, followed by a video-assisted thoracoscopy surgery to drain the hemothorax.

**Discussion:**

The bleeding dissected to both pleural cavities, causing the bilateral hemothorax. Although the patient did not have a medical diagnosis of neurofibromatosis before the occurrence, the presence of clinical signs of the disease, associated with the exclusion of other causes for hemothorax, allowed differential diagnosis and appropriate treatment.

**Conclusion:**

The differential diagnosis of neurofibromatosis should be advanced in cases of spontaneous bleeding. In patients diagnosed with neurofibromatosis, the risk of spontaneous bleeding due to the possibility of aneurysmal formation should be considered.

## Highlights

• In patients with NF1, the risk of spontaneous bleeding due to the possibility of aneurysmal formation should be considered.
• The differential diagnosis of neurofibromatosis should be investigated in cases of spontaneous hemothorax.
• In most cases of spontaneous hemothorax associated with NF1, intercostal artery aneurysm is the most common injury.

## 1 Introduction

There are four types of neurofibromatosis, and the most common are types 1 and 2. Type 1 neurofibromatosis (NF1), or von Recklinghausen's disease, is an autosomal dominant disease, related to a mutation in a gene on chromosome 17. It has an incidence of about 1 in 3.000 births. Usually diagnosed in adulthood, it can affect any organ system, especially connective, nervous and vascular tissues [[1], [2], [3], [4], [5], [6], [7], [8], [9], [10]].

Manifestations include cutaneous neurofibromas, café-au-lait spots [[2], [3], [4]], Lisch nodules, abnormal osseous lesions, macrocephaly [3] and tumors [1]. Vascular lesions associated with NF1 are rare (3,6% of the patients), including aneurysm and stenosis [4].

We present a case of bilateral hemothorax caused by the rupture of the right internal thoracic artery aneurysm in a patient with NF1.

This work has been reported in line with the SCARE criteria [5].

## 2 Presentation of a case

A 43-year-old man, with no past medical history but a family history of neurofibromatosis, was in his usual state of good health until he presented with sudden respiratory distress. He was admitted to the local health service, where his respiratory status deteriorated and endotracheal intubation was needed. Due to a high neck circumference, intubation was difficult and the patient presented continuous hypoxia until successful intubation was finally made after multiple attempts.

He was sent to our hospital after intubation. We admitted the patient hemodynamically stable, sedated, with no need of vasopressors. Physical examination revealed dullness to percussion, decreased breath sounds in both lung fields, multiple cutaneous nodules, café-au-lait spots and Lisch nodules. Thus, we diagnosed type 1 NF1 by clinical criteria and confirmed afterward with the biopsy of one of the skin lesions.

On admission, the hemoglobin level was 11.0 mg/dL and then decreased to 7,0 mg/dL in 12 h. Chest computed tomography (CT) with contrast revealed large bilateral hemothorax. Then, bilateral chest drainage was performed and approximately 600 mL of blood was evacuated on the left side, and 800 mL on the right side. Therefore, the patient received isotonic crystalloid and blood transfusion.

**Fig. 1:**
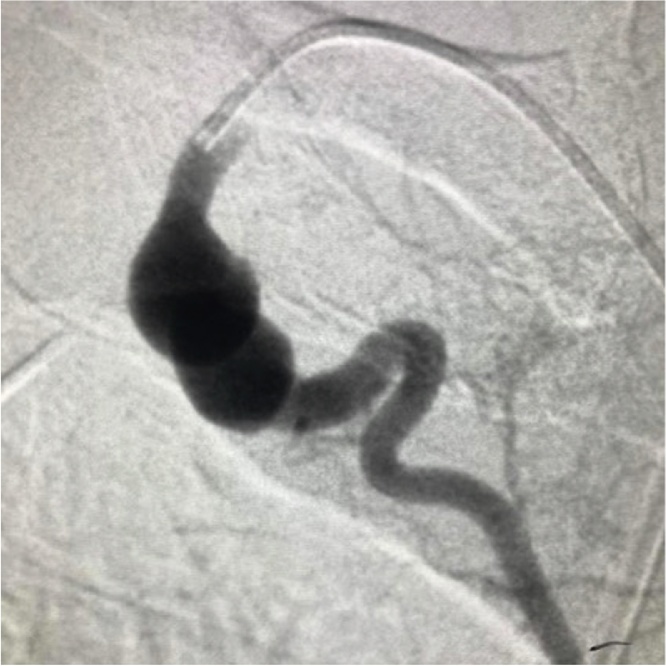
Right subclavian angiogram reveals an aneurysm in the proximal portion of the internal thoracic artery that originated from the right subclavian artery.

**Fig. 2:**
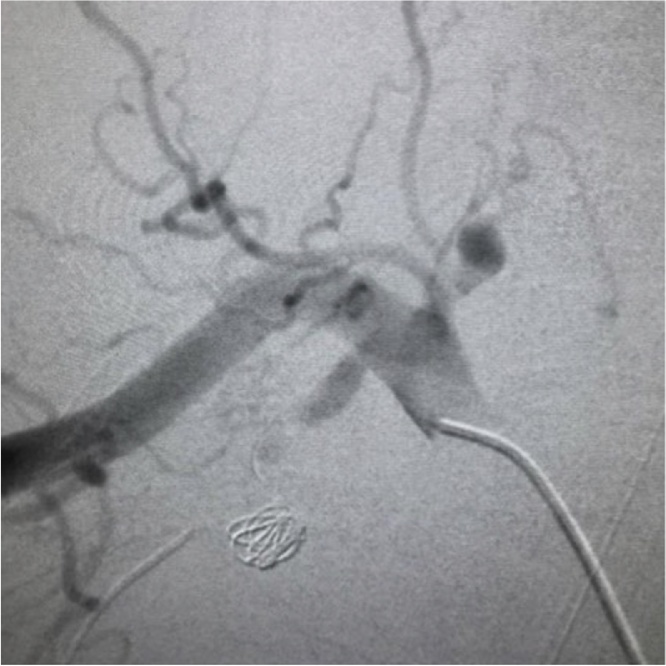
Right subclavian angiography after embolization shows complete occlusion of the right internal thoracic artery aneurysm with coils.

He was then submitted to arteriography, which evidenced a right internal thoracic artery aneurysm that was the probable source of the bleeding. However, there was no active bleeding or contrast leak. The aneurysm was selectively catheterized and embolized with coil (Fig. 1, Fig. 2), followed by a video-assisted thoracoscopy surgery (VATS) to drain the hemothorax, expand the lungs and review the pleural cavity. VATS showed retained and fibrous clot, suggesting chronic bleeding.

Despite therapeutic success in the first 48 h after the intervention, because of the hypoxia during initial intubation, the patient developed cerebral edema that culminated in brain death.

## 3 Discussion

NF1 is a multisystem disorder, and there are thoracic manifestations related to neurofibromas in mediastinum, chest wall, lateral thoracic meningocele and vascular structures. Moreover, other manifestations include dysplastic abnormalities of bones and the nervous system [6] and manifested vascular involvement as aneurysm, affecting approximately 3% of the patients [2,3].

Vascular lesions associated do NF1 include stenosis, occlusion, aneurysm, pseudoaneurysm, and rupture [7]. The mechanisms differ according to the size of the vessel: In large vessels, aneurysms are formed by the direct invasion of the vascular wall by tumors tissue, which presses the vasa vasorum and causes ischemia and weakness. Smaller vessels suffer dysplasia, that initiates stenosis of the wall, weakens and causes friability [2].

Only 30 cases of hemothorax associated with NF1 have been reported in the literature since 1982 in the English language. The arteries involved were: intercostal artery (33.3%), subclavian artery (10%), internal mammary artery (10%), extracranial vertebral artery (10%), pulmonary artery (3.3%), cervical artery (3.3%), thyrocervical trunk (3.3%), and in 20% of the reported cases, the vessel involved was not described. In only 6.7% (2 cases), the internal thoracic artery was involved [8,9].

In this case report, the classical cutaneous findings suggested the diagnosis hypothesis of NF1 for the patient. When there is a vasculopathy, signs and symptoms vary depending on the size and on the location of the lesion [2]. This patient had an acute onset of dyspnea and the increase of the cervical volume, due to the hematoma. However, initial symptoms could not be better investigated because the patient was admitted at the hospital already unconscious.

In this case, we found bilateral hemothorax in chest CT, but no suggestive etiology for the bleeding. Arteriography, on the other hand, showed an aneurysm in the right internal thoracic artery. The cause of the bilateral hemothorax was considered to be chronic bleeding of this aneurysm, explained by its degeneration and fragility, due to NF1. The bleeding dissected to both pleural cavities caused the bilateral hemothorax and impaired ventilation chronically. Chest CT was carefully interpreted looking for neurofibromas which could also be a cause of the hemothorax, as suggests previous case report, six but there were no other findings.

Endovascular embolization is indicated if the patient is hemodynamic stable. In unstable patients, it is recommended aggressive treatment, thoracotomy with surgical ligation [2,4]. VATS is believed to be the best available modality for the management of clotted hemothorax [4]. The treatment of massive hemothorax is by thoracotomy or VATS, to identify the cause and solve it. The surgical vessel reconstruction is limited because the arteries of patients with NF1 are fragile [10]. Previous published cases suggest that coil embolization has better results [2,9,10].

As the patient of this case was stable, the medical team opted to perform an endovascular embolization of the aneurysm and then use VATS to drain the pleural cavities and expand the lungs. VATS, then, showed retained and fibrous clot, suggesting chronic bleeding. The aneurysm embolization was effective in preventing new bleeding.

However, even with successful endovascular intervention and pulmonary expansion, the patient had a hypoxic-ischemic cerebral injury and died. It must be remembered that these are critical patients, and hemostasis does not always mean the resolution of the problem, once there can be many associated complications. The disease mortality is 36% and postoperative mortality is 33% in patients with hemothorax associated with NF [2].

## 4 Conclusion

The differential diagnosis of neurofibromatosis should be advanced in cases of spontaneous hemothorax. In patients diagnosed with neurofibromatosis, the risk of spontaneous bleeding due to the possibility of aneurysmal formation should be considered.

## Sources of funding

No sponsor involvement in the study.

## Ethical approval

The research was approved by the Research Ethics Committee of the Federal University of Vale do Sao Francisco protocol number 3649509 with the consent of University Hospital of the same university.

## Consent

Written informed consent was not obtained from the patient. The head of our medical team has taken responsibility that exhaustive attempts have been made to contact the family and that the paper has been sufficiently anonymised not to cause harm to the patient or their family. A copy of a signed document stating this is availble for review by the Editor-in-Chief of this journal on request.

## Author contribution

Antonio Felipe Neto: Writing Original Draft.

Edson Gonçalves Ferreira Junior: Review & Editing, investigation.

Larissa de Melo Freire Golveia Silveira: Review & Editing, investigation.

Felipe Gusmão: Data Curation, Investigation.

Karen Ruggeri Saad: Writing - Review & Editing.

Paulo Fernandes Saad: Supervision.

## Registration of research studies

The Plataforma Brasil is a database for recording human research. Our search has the following record: CAEE: 20876719.5.0000.5196.

## Guarantor

Paulo Fernandes Saad.

Head of residency in cardiovascular surgery.

## Provenance and peer review

Not commissioned, externally peer-reviewed.

## Declaration of Competing Interest

There is no conflict of interest.
